# Swelling Studies of Porous and Nonporous Semi-IPN Hydrogels for Sensor and Actuator Applications

**DOI:** 10.3390/mi11040425

**Published:** 2020-04-18

**Authors:** Daniela Franke, Gerald Gerlach

**Affiliations:** Institute of Solid State Electronics, Technische Universität Dresden, 01062 Dresden, Germany; gerald.gerlach@tu-dresden.de

**Keywords:** semi-interpenetrating network (IPN), porous hydrogel, bisensitive hydrogel, PNIPAAm

## Abstract

In this article, we present a semi-interpenetrating network (IPN) hydrogel of reasonable size with improved swelling behavior. The semi-IPN is composed of *N*-isopropylacrylamide and 2-acrylamido-2-methyl-1-propanesulfonic acid. Porosity was generated chemically by a surfactant-based template method. The swelling behavior was measured after an abrupt change of the temperature to 25 °C or 40 °C or after an abrupt change of the salt concentration of the aqueous medium surrounding the hydrogel samples. A set of static swelling degrees was determined from swelling measurements in salt solutions of varying concentrations and at different temperatures. Introducing porosity to the semi-IPN decreases the swelling times for most measurements while the sensor and actuator characteristics of the hydrogel found in previous studies are preserved. Additionally, we propose theoretical assumptions and explanations regarding the differences in the swelling kinetics of the porous and the nonporous semi-IPN and deduce implications for sensor and actuator applications.

## 1. Introduction

In this work, we study the benefits of introducing porosity to a semi-interpenetrating network (IPN) hydrogel designed for an application in an intramolecular force-compensated sensor as described in [1].

Hydrogels are three-dimensional organic polymer networks. Their ability of uptaking enormous amounts of water leads to a drastic increase of the hydrogel’s volume and is the reason for the widespread use in sanitary products. Stimuli-responsive hydrogels additionally contain one or more functional group/s, which react with a certain type of analyte or measurand being salt, acid/base, organics, temperature, electrical field, magnetic field or even light [2,3,4,5,6,7]. The functional groups may be incorporated in the main chain (or backbone) of the polymer (e.g., polyethylene oxid, polyethylene glycole) or they may be side groups or chains (e.g., polyacrylamide, polyacrylic acid, poly-*N*-isopropylacrylamide). This reaction normally is a change (decrease or increase) of the volume of the hydrogel depending on the change of analyte concentration or measurand value, respectively. The swelling and deswelling mechanisms are the result of the preference of the polymer forming intramolecular hydrogen bonds or hydrogen bonds between polymer parts and solvent molecules. The former leads to water release from the hydrogel and hence, deswelling. The latter causes swelling due to water uptake into the hydrogel structures [7,8,9,10,11,12]. The interaction of ions of salt solutions (based on ionic strength), acids or bases with the ionic or dissociable functional groups of polyelectrolyte hydrogels contributes to the swelling or deswelling as well as the repulsion of the ionic groups [8,13,14,15,16]. The swelling and deswelling is attributed to the change of the osmotic pressure composed of the mentioned contributions [8]. The restoring force of the polymer network always counterbalances the swelling [8].

The possible use of hydrogels as transducers in sensor applications is obvious when additionally considering that the synthesis of the materials is often low-cost and they can be integrated into several different sensor setups [8,17]. In addition, the use of hydrogels as actuators in pumps, microvalves and as artificial skin was considered and carried out [18,19].

By IUPAC definition, a semi-interpenetrating polymer network (semi-IPN) is a polymer consisting of a chemically linked polymer network and a polymer chain. The polymer chain penetrates the polymer network and is therefore physically attached to it [20]. It can be referred to as a polymer blend. Studies on semi-IPNs and IPNs of different compositions have been reported [13,21,22,23]. In this work, we blended a salt- (ionic strength) and temperature-sensitive copolymer network made of 2-acrylamido-2-methyl-1-propanesulfonic acid (AMPS) and *N*-isopropylacrylamide (NIPAAm) with salt- (ionic strength-) sensitive poly-AMPS chains formally forming [*net*-poly(2-acrylamido-2-methyl-1-propanesulfonic acid-*co*-*N*-isopropylacrylamide)]-*sipn*-poly(2-acrylamido-2-methyl-1-propanesulfonic acid) [24] referred to as “semi-IPN” in the following text.

Previous work shown in [1] demonstrates that this composition of a semi-IPN showed proper swelling characteristics and mechanical stability for the use in the piezoresistive setup operating with closed-loop force-compensation. Hereby, the poly-AMPS (PAMPS) part of the hydrogel is the sensitive part while the poly-NIPAAm (PNIPAAm) part is an actuator applying the compensation force controlled by temperature between 25 °C and 50 °C. A comprehensive overview of the functionality of ionic strength and temperature sensitive hydrogels can be found in [12,25].

However, a tremendous amount of publications show that nonporous hydrogels, though sensitive to many analytes and measurands, feature very slow swelling kinetics depending on size and geometry of the sample leading to long response times of the sensor. A possible solution for this problem is the insertion of channel structures into the hydrogel body to ensure a faster transport of the analyte solution within the hydrogel and therefore accelerate the swelling answer. The positive effect was already shown by Schulz et al. who demonstrated a reduction in response time of about 80% (compared to a nonporous hydrogel) when using a porous pH sensitive hydrogel in a piezoresistive sensor setup [26]. In our approach, we form an interconnected porous network inside the semi-IPN hydrogel by chemical synthesis using a surfactant-based template method. We already introduced this method in [27] where we (in part) also showed a major decrease of the response time of a temperature sensor based on PNIPAAm. The synthesis method is described e.g., in [28].

In this work, we extend the synthesis method in [27] to the semi-IPN from [1] in order to receive a porous semi-IPN with enhanced swelling characteristics with respect to temperature and salt concentration.

## 2. Materials and Methods 

### 2.1. Materials

The polymer poly(2-acrylamido-2-methyl-1-propanesulfonic acid) (in solution, 15 wt.% in water) (PAMPS), the monomers 2-acrylamido-2-methyl-1-propanesulfonic acid (AMPS), *N*-isopropylacrylamide (NIPAAm), the crosslinker *N,N′*-methylenebis(acrylamide) (MBA), the initiator system consisting of ammonium persulfate (APS) and *N,N,N′,N′*-tetramethylethylenediamine (TEMED), the surfactant Brij L23, sodium hydroxide and sodium chloride were bought from Sigma Aldrich (Munich, Germany) and were used as received.

### 2.2. Syntheses

**Scheme 1:**
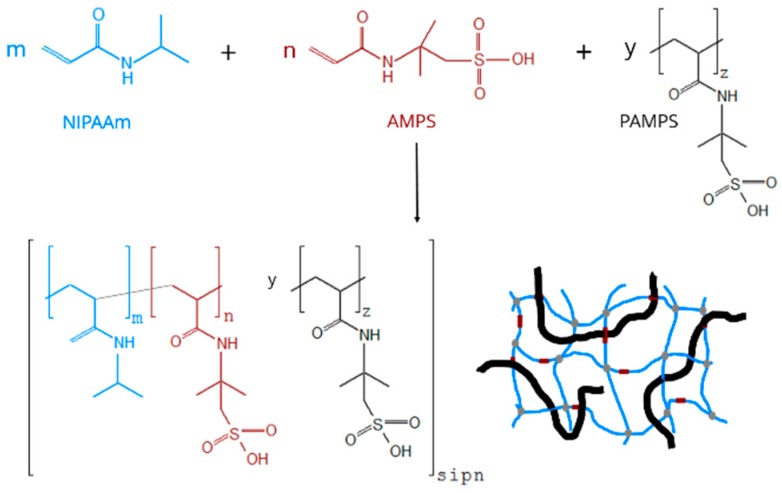
Reaction mechanism of the synthesis and schematic representation of the semi-interpenetrating network. Colors of the schematic representation mean: (blue) poly-*N*-isopropylacrylamide (PNIPAAm), (brown) poly-2-acrylamido-2-methyl-1-propanesulfonic acid (PAMPS) from 2-acrylamido-2-methyl-1-propanesulfonic acid (AMPS) monomers, (black) PAMPS chains, (grey) chemical cross-links.

To prepare the nonporous semi-IPN, 0.3677 g PAMPS solution (equals 0.27 mmol AMPS), 0.0601 g (0.29 mmol) AMPS, 1.0595 g (9.36 mmol) NIPAAm and 0.0593 g (0.38 mmol) MBA were dissolved in 7.98 g deionized water. The pH value of this acidic solution was increased to 7 by adding 600 µL 1 M sodium hydroxide solution. The solution was stirred and degassed with nitrogen at 0 °C for 30 min. After that, 57.3 µL 0.84 M APS solution (equals 0.048 mmol APS) and 7.3 µL (0.048 mmol) TEMED were added to the previous solution under stirring. The transfer of this reaction solution to a cooled container (a capillary with an inner diameter of 4 mm and a length of 180 mm) was done instantly. The polymerization occurred at room temperature after several minutes. For preparing the porous semi-IPN, 0.473 g Brij L23 was added to a reaction solution of the composition as described above. The remaining procedure was carried out analogous. The syntheses result in semi-IPNs composed of 94.4 mole percent NIPAAm and 5.6 mole percent AMPS. The reaction mechanism is shown in Scheme 1.

After the reaction, the polymer cylinders were taken out of the capillaries and rinsed in deionized water for three days (nonporous hydrogel) and 30 days (porous hydrogel), respectively. After rinsing, the cylinders were cut into disks with a height of about 2 mm.

### 2.3. Scanning Electron Microscopy (SEM) Observation

The morphology of the hydrogels was pictured using scanning electron microscopy (SEM). Freeze-dried samples of both the nonporous and the porous hydrogel were put on adhesive carbon foils. Then, 3 nm of platinum were sputtered on the surface of the samples using a Leica EM SCD500 sputtering system (Leica Microsystems GmbH, Wetzlar, Germany). A Neon 40EsB scanning electron microscope from Carl Zeiss Microscopy GmbH (Jena, Germany) with a Schottky field-emission electron source and an extra high tension (EHT) voltage level of the electron beam of 3 kV was used to determine the SEM pictures. Images were received from signals of a secondary electron (SE2) detector and an In-Lens detector located in the scanning electron microscope in standard setup. Only fracture surfaces were recorded in order to ensure the imaging of the inside of the hydrogel samples. Pore diameters were measured using the “particle analyze” tool of the ImageJ software package [29].

### 2.4. Swelling Kinetics

Swelling kinetics were studied by abruptly changing the temperature or salt concentration of the hydrogel-containing solution and plotting the change of the swelling degree over time by taking photographs in time lapse through a microscope setup. The resulting swelling degree is calculated as

(1) $$Q_{d}=\frac{d_{t}}{d_{0}},$$

where *Q_d_* is the swelling degree determined from the change of the disk’s diameter, *d_t_* is the diameter of the disk at a certain time of the measurement and *d_0_* is the disk’s diameter before the stimulus was applied. In the setup, the hydrogel disk sits in a copper well filled with water. The temperature of the bath is adjusted at 25 °C or 40 °C using a Peltier element, the HAT control temperature controller from BelektroniG GmbH (Freital, Germany) and the software BelektroniG HAT Soft Pro v2.3.23. The sodium chloride concentration of the solutions were 0 M (deionized water) or 0.01 M. Images were taken through a microscope using a Bresser Microcam Pro HDMI and the software Bresser MicroCamLab II. For the determination of the diameters of the hydrogel disks, we used the homemade Python-based software PIA [30]. Large standard deviations and deviations of the mean values from smooth curve characteristics in some of the following figures mainly result from these methods of determining and evaluating images not from the hydrogels themselves. Swelling and deswelling times were evaluated graphically by identifying the equilibrium swelling degree (plateau) and determining the time of its first occurrence.

### 2.5. Static Degrees of Swelling

After conditioning the hydrogel disks for two weeks, three samples each were put into sodium chloride solutions of the concentrations 0.0001 M, 0.00032 M, 0.001 M, 0.0032 M, 0.01 M, 0.032 M, 0.1 M, 0.32 M, 1 M. The solutions were stored at 25 °C for three days. The sodium chloride solution is changed once a day in order to ensure the complete exchange of water for the salt solution inside the hydrogel samples. The procedure was repeated for the temperatures 30 °C, 35 °C, 40 °C, 45 °C and 50 °C. The samples were weighed at each temperature step in order to evaluate the mass-based swelling degree *Q_m_*. The latter is calculated as

(2) $$Q_{m}=\frac{m\left(T,c\right)-m_{dried}}{m_{dried}},$$

where *m(T,c)* is the mass of the hydrogel sample depending on the temperature *T* and the salt concentration *c* and *m_dried_* is the mass of the freeze-dried hydrogel sample, a value that was collected after the described measurements took place. For the evaluation, mean values and standard deviations of *Q_m_* were calculated for samples from the same salt solution.

## 3. Results and Discussion

### 3.1. Scanning Electron Microscopy (SEM)

**Figure 1:**
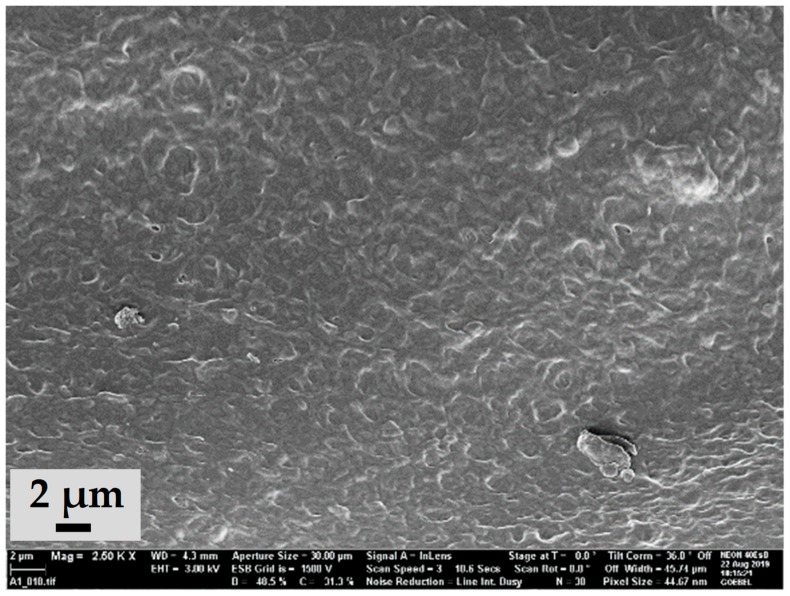
SEM image of nonporous semi-IPN.

The SEM image of the fracture surface belonging to the nonporous semi-IPN is shown in Figure 1. The hydrogel is solid and nonporous as expected.

**Figure 2:**
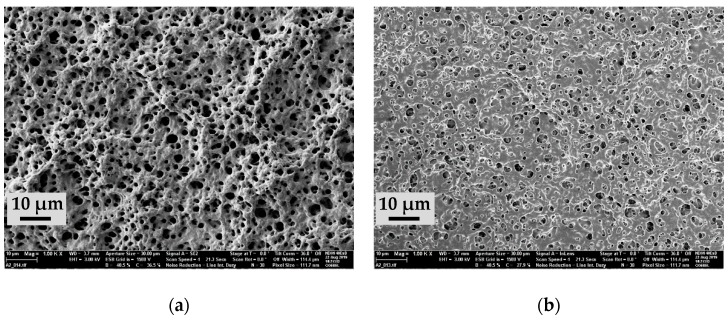
SEM images of the same position on the fracture surface of the porous semi-IPN obtained from (**a**) the secondary electron (SE2) detector; (**b**) the In-Lens detector.

In contrast, the SEM images from a fracture surface of the porous semi-IPN clearly show porosity (Figure 2). The image received from the SE2 detector (Figure 2a) shows the topology of the sample with circular pore openings uniformly distributed over the fracture surface. Catching these secondary electrons with the In-Lens detector gives an image with more depth (Figure 2b) showing open porosity through the outer pores, and thus, providing the channel system in the hydrogel as required for faster swelling kinetics.

**Figure 3:**
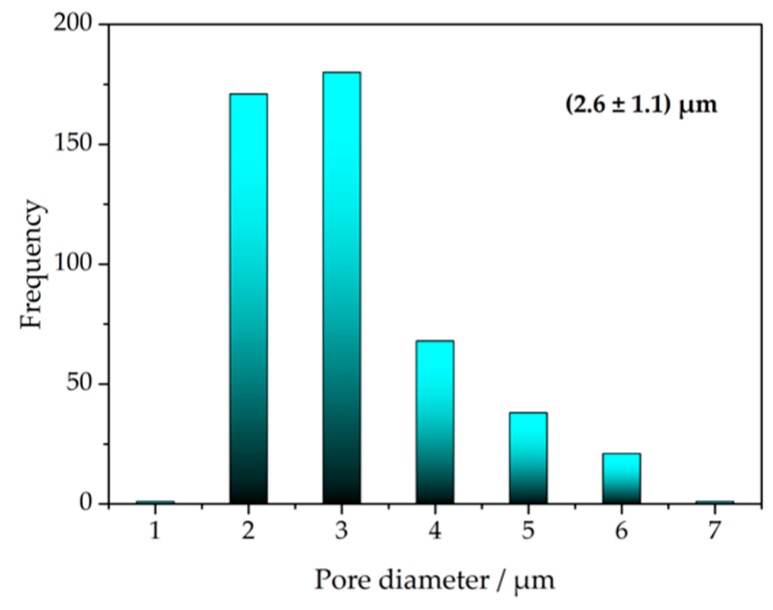
Distribution of pore diameters of the porous semi-IPN.

The pore diameters were determined from Figure 2a. Figure 3 shows the distribution of the pore diameters. The average pore diameter is 2.6 µm with a standard deviation of 1.1 µm. It is therefore similar to the value found for porous PNIPAAm in [27] showing the transferability of the synthesis method to other more complex hydrogel systems.

### 3.2. Swelling Kinetics

#### 3.2.1. Temperature-Dependent Swelling Behavior

**Figure 4:**
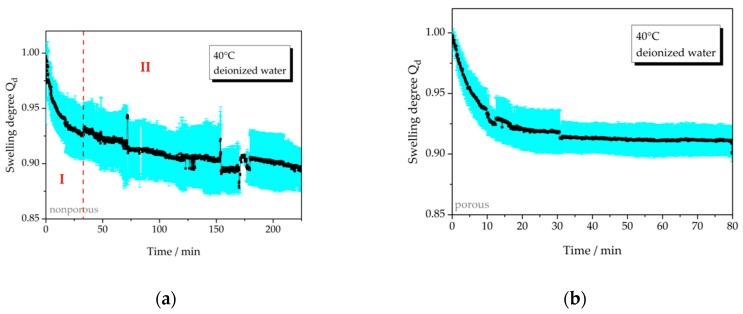
Comparison of the deswelling kinetics of (**a**) a nonporous and (**b**) a porous semi-IPN hydrogel. The measurements were carried out at 40 °C after abruptly changing the temperature from 25 °C to 40 °C. Black squares refer to average values. The standard deviations are pictured in light blue and appear as a cloud due to the huge amount of measurement values.

Temperature-depending deswelling of the nonporous and the porous semi-IPN is shown in Figure 4 when abruptly changing the temperature from 25 °C to 40 °C. This behavior is caused by the PNIPAAm part of the semi-IPN hydrogel. It is obvious from Figure 4 that the porous hydrogel (Figure 4b) deswells much faster than the nonporous one (Figure 4a). The equilibrium swelling degree (constant value) is reached after 20 min while the nonporous hydrogel does not reach the equilibrium swelling value. This agrees with the findings in [27] where we found a significant decrease of the response time of a piezoresistive microsensor when using a porous PNIPAAm hydrogel as the transducer. The deswelling times found for PNIPAAm in [27] are much lower because the hydrogel sample used there is much smaller. The swelling time depends on the size and geometry of the hydrogel sample.

Additionally, a pronounced so-called skin effect is found for the nonporous hydrogel leading to a two-step behavior of the deswelling curve as demonstrated in Figure 4a by the red vertical line. In section I of the deswelling curve the near-surface part of the hydrogel disk deswells comparably fast due to short diffusion distances for the transport of water to the outside of the hydrogel. During this process, this part of the hydrogel collapses and forms a hydrophobic barrier for the transport of water from the inner part of the hydrogel disk causing very slow release of water and, hence, a long deswelling time represented in section II of the deswelling curve. (Nayak et al. [12] neatly described the temperature-dependent phase transition behavior of PNIPAAm.)

The porous hydrogel does not show this skin effect leading to the assumption that the open channel structure inside the hydrogel provides transport paths for water through the hydrophobic barrier. This is an important new feature of the semi-IPN because up to now the skin effect is a major drawback for the use of PNIPAAm as transducers in sensors and as actuators and is normally hard to overcome by downsizing the hydrogel piece to µm or nm dimensions making the hydrogel less easy to handle. Instead, here, we present a hydrogel sample of reasonable size with improved deswelling behavior.

**Figure 5:**
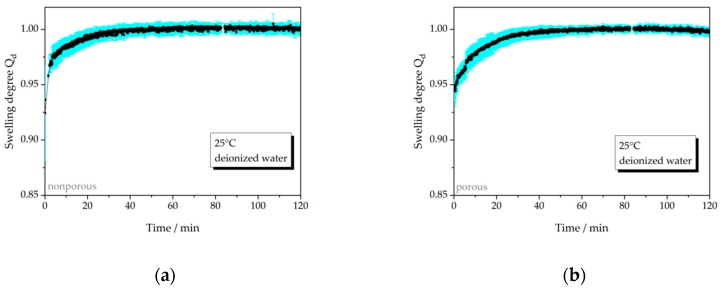
Comparison of the swelling kinetics of (**a**) a nonporous and (**b**) a porous hydrogel sample. The measurements were carried out at 25 °C after abruptly changing the temperature from 40 °C to 25 °C.

Figure 5 shows the swelling behavior of the hydrogel samples when changing the temperature from 40 °C to 25 °C. Here, the nonporous semi-IPN needs 50 min to swell to the equilibrium swelling degree while the porous semi-IPN needs 45 min and therefore, there is no significant difference between the swelling times. In addition, compared to Figure 4b the swelling time for the porous semi-IPN is much slower than the deswelling time. A possible explanation for this behavior might be a significant decrease of pore diameters or the absence of pores in the deswollen state at 40 °C leading to nonporous behavior and explaining similarity of the swelling times in Figure 5a,b.

For easy comparison, the swelling degrees and times are summarized in Table 1.

#### 3.2.2. Swelling Behavior Depending on Salt Concentration

As described in [12], the PNIPAAm hydrogel is hydrophilic below the critical temperature of 32 °C (LCST, lower critical solution temperature) and turns hydrophobic above this temperature. We expect the same behavior for the PNIPAAm part in the semi-IPN turning a major part of the semi-IPN hydrophobic at 40 °C. Hydrophobicity of the polymer network should hinder the uptake of water at swelling conditions in deionized water and cause long swelling times compared to faster swelling in a hydrophilic network at 25 °C. The same can be presumed for the deswelling in a salt solution where sodium and chloride ions cause the release of water from the hydrogel due to the osmotic pressure and ion exchange. Water has to be released from the hydrogel through the hydrophilic (at 25 °C) or the hydrophobic (at 40 °C) network. Hence, in the following, we show the deswelling (in 0.01 M NaCl solution) and the swelling (in deionized water) behavior at a temperature below 32 °C and a temperature above 32 °C in order to study the influence of hydrophilic and hydrophobic properties of the semi-IPN on the rate of uptake and release of water.

**Figure 6:**
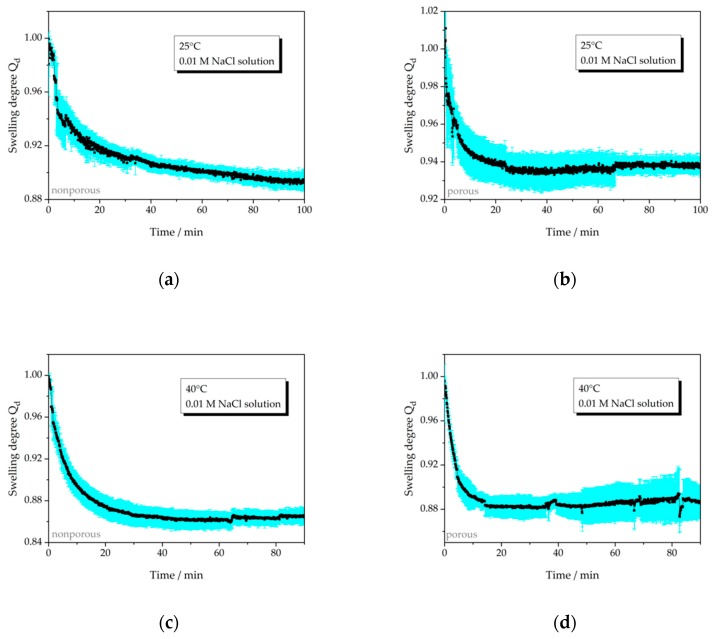
Comparison of the deswelling kinetics of (**a**), (**c**) a nonporous semi-IPN hydrogel and (**b**), (**d**) a porous semi-IPN hydrogel at temperatures (**a**), (**b**) 25 °C and (**c**), (**d**) 40 °C. The measurements were carried out in 0.01 M NaCl solution after abruptly changing the aqueous medium from deionized water to 0.01 M NaCl solution.

Figure 6 compares the deswelling behavior of the nonporous and the porous semi-IPN in 0.01 M NaCl solution at 25 °C and 40 °C. At 25 °C (Figure 6a,b), a deswelling time of 25 min is found for the porous hydrogel while the nonporous hydrogel has not reached the equilibrium swelling degree during the measurement period. Again, this faster deswelling of the porous hydrogel can be explained with the open channel structure increasing the rate of transport of the sodium ions, chloride ions and water inside the hydrogel. In contrast, the difference of deswelling times is smaller at 40 °C. The nonporous hydrogel needs 43 min to reach the equilibrium swelling degree while the porous semi-IPN needs about 15 min. We discuss this difference in the swelling behavior at different temperatures in Section 3.2.3.

**Figure 7:**
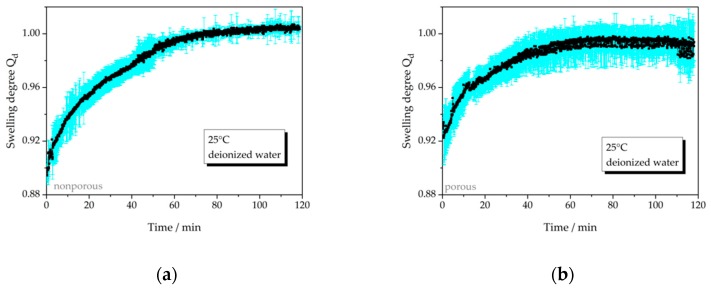

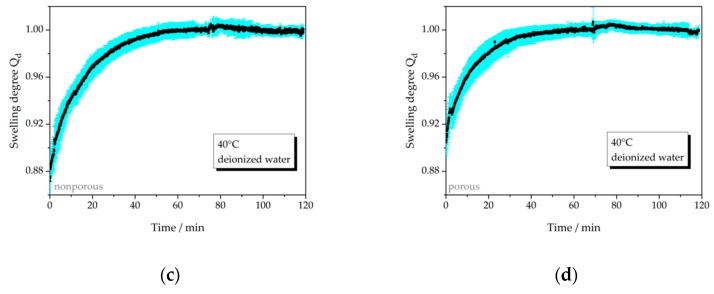
Comparison of the swelling kinetics of (**a**), (**c**) a nonporous semi-IPN hydrogel and (**b**), (**d**) a porous semi-IPN hydrogel at temperatures (**a**), (**b**) 25 °C and (**c**), (**d**) 40 °C. The measurements were carried out in deionized water after abruptly changing the aqueous medium from 0.01 M NaCl solution to deionized water.

Figure 7 compares the swelling of the nonporous and the porous semi-IPN after abruptly changing the aqueous medium from 0.01 M NaCl solution to deionized water at 25 °C and 40 °C, respectively. While the nonporous semi-IPN does not reach the equilibrium swelling degree at 25 °C the porous one completely swells in 60 min as expected. At 40 °C, the difference between the swelling times of nonporous (60 min) and porous (50 min) semi-IPN is not highly pronounced. We explain this behavior in Section 3.2.3.

Table 2 summarizes the measured swelling degrees and swelling times for the swelling and the deswelling of the hydrogels.

#### 3.2.3. Discussion of Deswelling and Swelling Mechanisms in Salt Solution

For the explanation of differing deswelling and swelling behavior of nonporous and porous semi-IPNs we consider the following mass transport mechanisms:

(1) Mass transport of water based on osmotic pressure,
(2) Mass transport of sodium and chloride ions based on the Gibbs–Donnan effect,
(3) Water transport based on ionic exchange at the ionic groups of the hydrogel (rules: smaller H^+^ ions are exchanged for larger Na^+^ ions, and larger ions have a smaller hydration shell),
(4) Diffusion of sodium and chloride ions according to Fick‘s law based on a concentration gradient and
(5) Convection.

We assume that these mass transport mechanisms occur with varying magnitude and, hence, affect the swelling behavior differently resulting in different swelling times at different conditions. 

##### Deswelling

**Figure 8:**
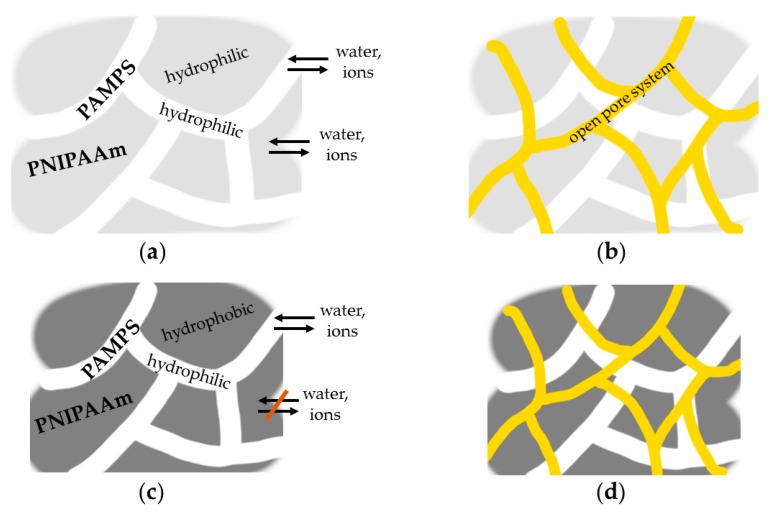
Schematic illustration of transport pathways and swelling/deswelling mechanisms for (**a**), (**c**) the nonporous semi-IPN and (**b**), (**d**) the porous semi-IPN at (**a**), (**b**) 25 °C and (**c**), (**d**) 40 °C. The arrangement of the pictures corresponds to Figure 6 and Figure 7 and explains our basic interpretation of the swelling and deswelling behavior in salt solution at different temperatures. The colors refer to (white) the PAMPS part, (light grey) the hydrophilic PNIPAAm part (at 25 °C), (dark grey) the hydrophobic PNIPAAm part (at 40 °C) and (yellow) the pore system.

The hydrogel sample pictured schematically in Figure 8a refers to the measurement pictured in Figure 6a and is completely hydrophilic, but only the white PAMPS parts, that have a small share of 5.6 mole percent in the whole hydrogel sample, are able to deswell when put in the salt solution. For these parts the transport mechanisms (1), (2) and (3) are decisive. The difference in activities of the aqueous media inside and outside of the hydrogel defines the osmotic pressure and causes a mass transport of water to the outside of the hydrogel. The diffusion of the ions to form the Gibbs–Donnan equilibrium also causes the release of water due to mechanism (3). The Na^+^ ions are exchanged for protons (H^+^ ions) at the sulfonic acid groups. Due to a smaller hydration shell of Na^+^ ions and a therefore increased attraction of the negatively charged sulfonic acid groups to the sodium ions, the ions and ionic groups move closer together and, hence, water is released from the hydrogel.

For the also hydrophilic PNIPAAm part, the mechanisms (1) and (4) are relevant. The explanation for (1) is the same as described above. Mechanism (4) describes the diffusion of ions into the hydrophilic PNIPAAm part based on a concentration gradient. Since this part of the hydrogel is nonionic there is no attraction of ions to this hydrogel part and, hence, no compression forced by inner interaction. Physical reactions like van der Waals reactions of the polymer chains with the Na^+^ and Cl^−^ ions do not happen. Hence, only the PAMPS part is capable of deswelling, PNIPAAm has to be compressed by the smaller PAMPS part meaning that water has to be pressed out of the uninvolved larger PNIPAAm part while, due to mechanism (4), a mass transport of ions to the inside of the PNIPAAm part occurs. This procedure is bound to take a long time considering the long deswelling time for PNIPAAm under temperature-induced deswelling conditions as presented in Figure 4a.

However, in Figure 6c and Figure 8c at 40 °C only the PAMPS part of the hydrogel is hydrophilic. The already compressed PNIPAAm part is hydrophobic and, therefore, less accessible for ions and water. Hence, no mass transport in PNIPAAm parts is expected neither from the inside nor from the outside of the hydrogel. The transport mechanisms in the PAMPS parts are the same as in Figure 8a. Hence, there are much less transport pathways for the release of water and the diffusion of ions and a longer deswelling time would be expected, but in the experiment the deswelling is much faster. Obviously, the major time-increasing part of the deswelling mechanism is to force the deswelling of PNIPAAm under non-deswelling conditions. When this part is already deswollen this transport step is omitted. It seems that at 40 °C a major part of the mass transport is caused by osmotic pressure (mechanism (1)) because it does not seem plausible that only the mass transport of ions (mechanisms (2, 4)) to the inside of an almost completely hydrophobic hydrogel contributes much to the decrease of the deswelling time. These transport paths are restricted. On the other hand, at 25 °C, the osmotic pressure (mechanism (1)) plays a minor role in the deswelling because the immediate diffusion of ions caused by mechanisms (2) and (4) to the inside of the semi-IPN balances the ion concentrations and, hence, the chemical potentials of the aqueous media inside and outside of the hydrogel. The time-limiting step of the deswelling process is the necessity of decompressing the uninvolved PNIPAAm parts of the semi-IPN by the PAMPS parts.

The deswelling of the porous semi-IPN is much faster at both temperatures. We explain this with the existence of the open pore system as pictured in yellow in Figure 8b,d. Mass transport mechanism (5) seems therefore to be predominant. Here, also the hydrophobic, already mainly deswollen semi-IPN (at 40 °C) deswells faster than the hydrophilic semi-IPN at 25 °C. This is explained in the same way as for the nonporous semi-IPN.

##### Swelling

For the deswelling in salt solution, we assume that the transport mechanisms (1), (2), (4) and (5) in Figure 8a–d occur but oppositely directed. However, based on the rules for the mechanism (3) we do not expect it to be relevant for the swelling. The mechanism of ionic exchange is replaced by considerations of entropy. The water that the hydrogel takes up, due to (1), (2) and (5), dilutes the salt solution and, hence, increases the entropy inside the hydrogel. Same goes for the water outside of the hydrogel mixing with Na^+^ and Cl^−^ ions coming from the inside of the hydrogel due to mechanisms (2), (4) and (5).

For the difference of the swelling times in Figure 7a,c, we propose the same explanation as for the difference in deswelling times in Figure 6a,c, but vice versa. The uninvolved PNIPAAm part of the semi-IPN has a major influence on the swelling time depending on its hydrophilic or hydrophobic state. The swelling times of the nonporous and porous semi-IPNs in Figure 7b,d are similar. It seems that, although the mass transport mechanisms have a different impact on the swelling kinetics, they lead to the same slow swelling times. Even porosity does not decrease the swelling time. Theoretic calculations especially including diffusion coefficients have to examine and can maybe explain this behavior.

### 3.3. Static Degrees of Swelling

**Figure 9:**
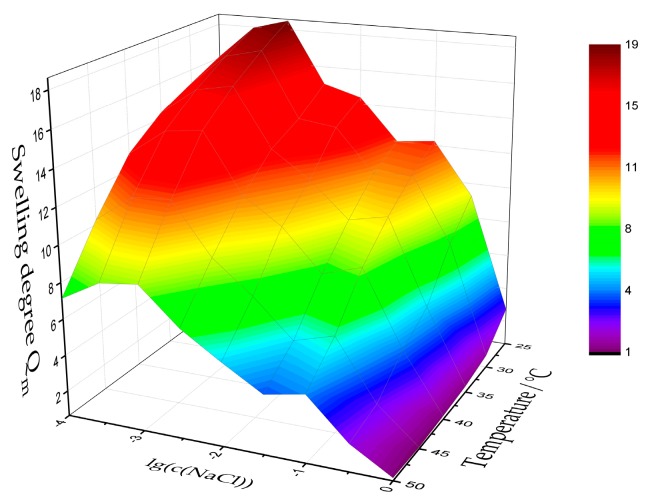
Static swelling behavior of the porous semi-IPN. The diagram shows the mass-based equilibrium swelling degree in dependence on both temperature and concentration of the sodium chloride solution.

Figure 9 shows the static swelling behavior of the porous semi-IPN. The results for the nonporous semi-IPN were already presented in [1]. The characteristics of the curve and the static swelling degrees are similar for both the nonporous and the porous hydrogel. This is expected because the “addition” of porosity to the semi-IPN should not alter the course of the dependencies. The swelling degrees can differ because the porous hydrogel contains less swellable material per volume unit. However, here, we did not find any differences.

In [1] the characteristics of the surface curve of the nonporous semi-IPN was found to be suitable for a force-compensated sensor application. The predefinition of parameter ranges (concerning temperature and salt concentration) is discussed there. Obviously, since the characteristics of the surface curve is similar when using the porous semi-IPN, its applicability is preserved regarding the use as a combined transducer-actuator in a force-compensated sensor.

**Figure 10:**
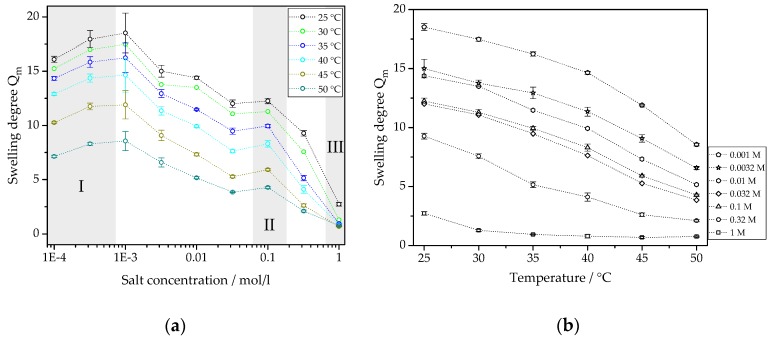
Characteristics for (**a**) the sensor part and (**b**) the actuator part of the semi-IPN. The data points and curves are a projection of the contour plots in Figure 9.

Figure 10 projects the data points from Figure 9 into two-dimensional coordinate systems. Figure 10a represents the sensor characteristics of the semi-IPN (view along the temperature axis in Figure 9), and Figure 10b depicts the curves of the actuator part (view along the concentration axis in Figure 9). The data points are first discussed in the shaded areas I to III in Figure 10a because they fall out of the expected behavior for several reasons. Area I shows that the detection limit is reached with the salt concentration of 0.001 M. Below this concentration no further swelling happens. The swelling degrees determined from 0.0001 M and 0.00032 M salt solutions vary within the uncertainty of the swelling degree from 0.001 M salt solution. In [1], the swelling degrees in this area of the surface curve are constant backing up this assumption. In Figure 10a, we assume that the swelling degrees are constant for low salt concentrations. Area II shows an unexpected peak in the curves of the swelling degree. Since this peak recurs for every temperature and we do not expect a sudden increase of the swelling degree for this concentration we assume that a mistake occurred when preparing the salt solution. For the concentration of 1 M (area III), obviously, the saturation point of the semi-IPN is reached. The hydrogel is completely deswollen and a change in temperature does not change the swelling degree any more. Only at 25 °C, a temperature clearly below the LCST, a small increase of the swelling degree occurs.

When leaving the data points in these shaded areas of Figure 10a out of the consideration, a linear relationship of swelling degree and the salt concentration (in logarithmic scale) occurs for each temperature. The slopes are very similar. Linearity of the sensor’s characteristic curves is important for a simple evaluation of the transducer signal without the necessity of additional signal processing.

Figure 10b shows the characteristic curves of the actuator part of the porous semi-IPN. These curves also show a linear behavior. Only the curves at lower and higher concentrations deviate from linearity. In addition, as described for Figure 10a, the swelling degree at a salt concentration of 1 M does not depend on the temperature anymore. Linearity of the characteristic curves is helpful for the control and activation of the actuator in the force-compensated sensor.

We find these linear characteristics to be an improvement due to the combination of the monomers AMPS and NIPAAm in a copolymer when usually the static swelling behavior of PNIPAAm is found to be discontinuous [31].

## 4. Conclusions

With regard to sensor and actuator applications of the porous semi-IPN positive results could be achieved. We found linearity for both the sensor and the actuator characteristics. The transducer signal can be evaluated easily to provide the sensor signal. The actuator characteristics could be changed from a discontinuous to a linear behavior by modifying the chemical composition of the semi-IPN. In most swelling measurements the porosity of the semi-IPNs decreases the swelling times significantly while not changing the other positive sensor and actuator characteristics found for nonporous semi-IPN.

However, although overcoming most obstacles occurring from nonporosity and unsuitable hydrogel composition, we still have to consider the chemical characteristics of the different parts of the semi-IPN. Although the actuator characteristics were linearized by modifying the chemical composition of the hydrogel, the interplay of hydrophilicity and hydrophobicity still plays a major role for the swelling kinetics. The LCST at 32 °C is still relevant for the PNIPAAm part of the hydrogel, although it cannot be seen in the diagrams for static swelling degrees. Discontinuous behavior might still occur for the swelling and deswelling times. This has to be investigated in further studies.

The assumptions and explanations in Section 3.2.3 have the quality of hypotheses. The mentioned mass transport mechanisms and associated theories are widely used for the modeling and simulation of hydrogel swelling behavior. The plausibility of our assumptions has to be proved in further studies and calculations. An accurate verification of experiments with calculations and vice versa is important and has to be expanded in the future.

## Figures and Tables

**Table 1:** Results of the deswelling and swelling after temperature changes; np, nonporous, p, porous.

Swelling Conditions	Figure	∆Q	t/min
40 °C, np	4a	>0.10	−
40 °C, p	4b	0.08	20
25 °C, np	5a	not relevant	50
25 °C, p	5b	not relevant	45

**Table 2:** Results of the deswelling and swelling in salt solution or water at different temperatures; np, nonporous, p, porous.

Swelling Conditions	Figure	∆Q	t/min
25 °C, np, salt	6a	>0.10	−
25 °C, p, salt	6b	0.06	25
40 °C, np, salt	6c	0.14	43
40 °C, p, salt	6d	0.12	15
25 °C, np, water	7a	not relevant	−
25 °C, p, water	7b	not relevant	60
40 °C, np, water	7c	not relevant	60
40 °C, p, water	7d	not relevant	50
